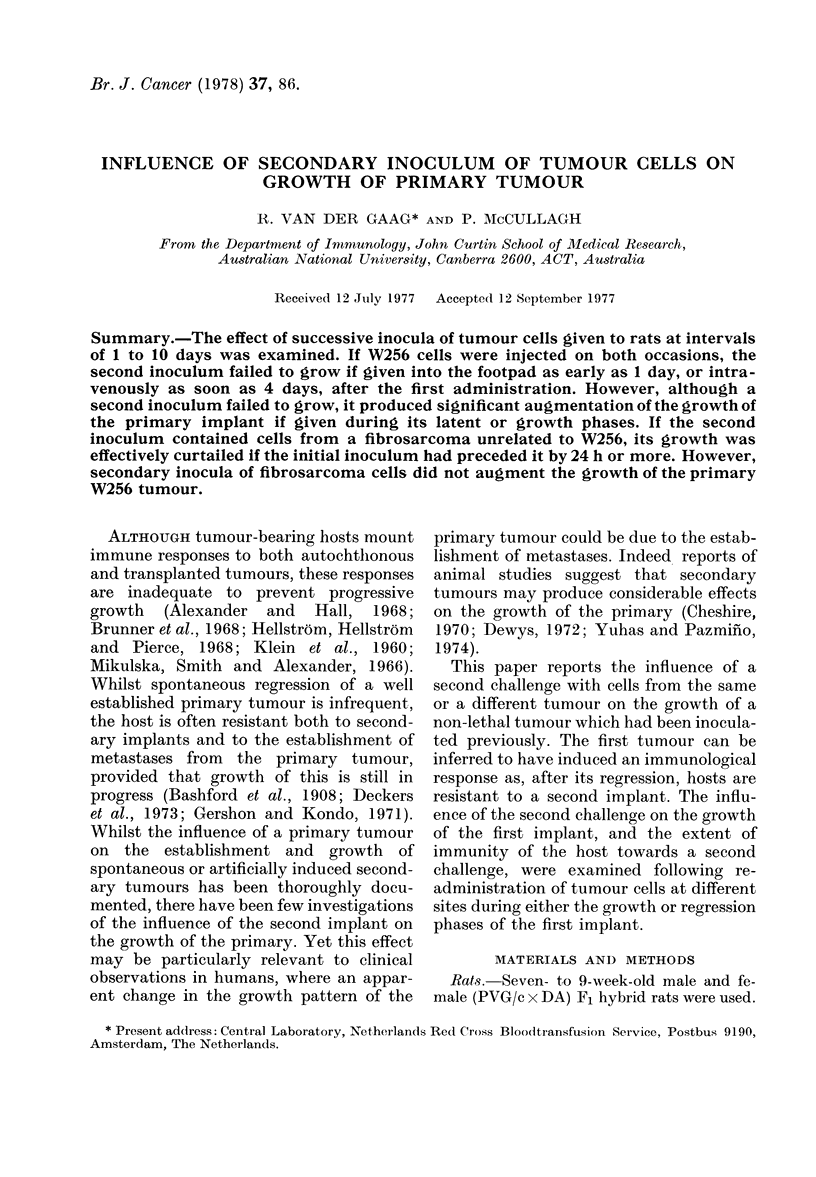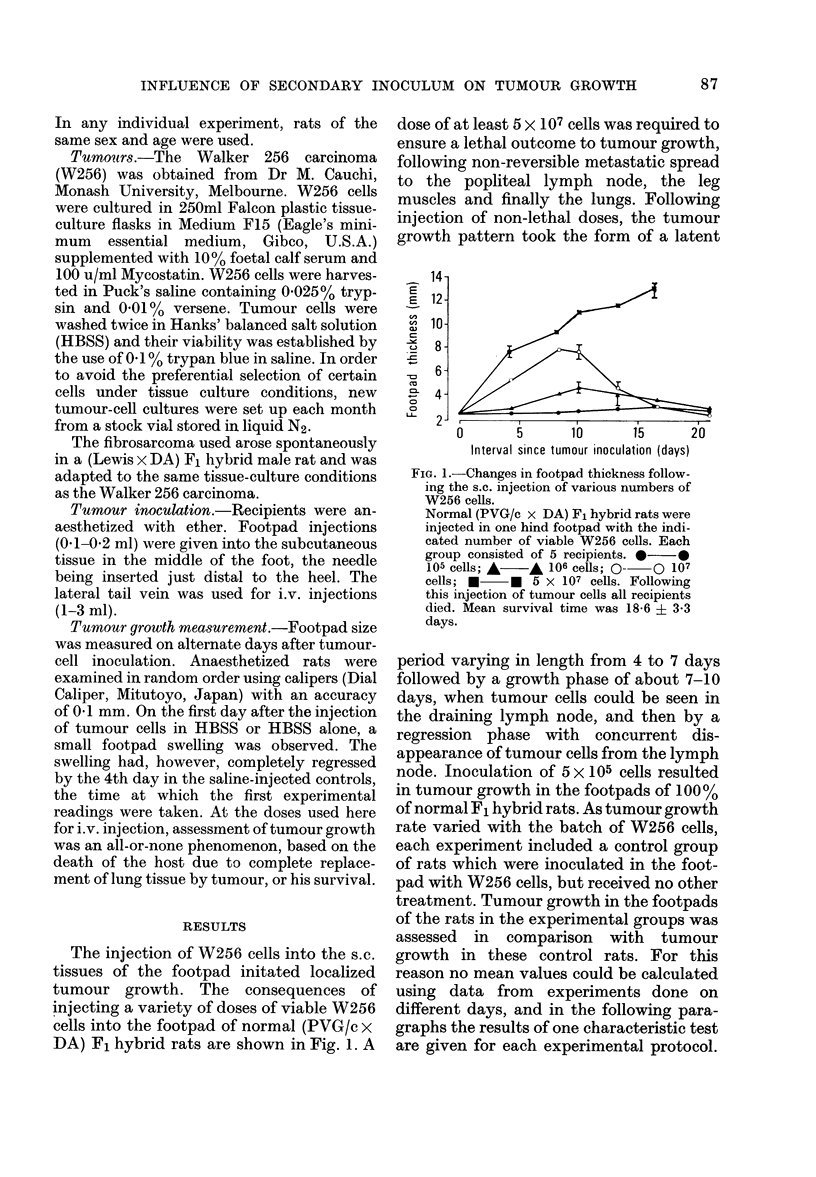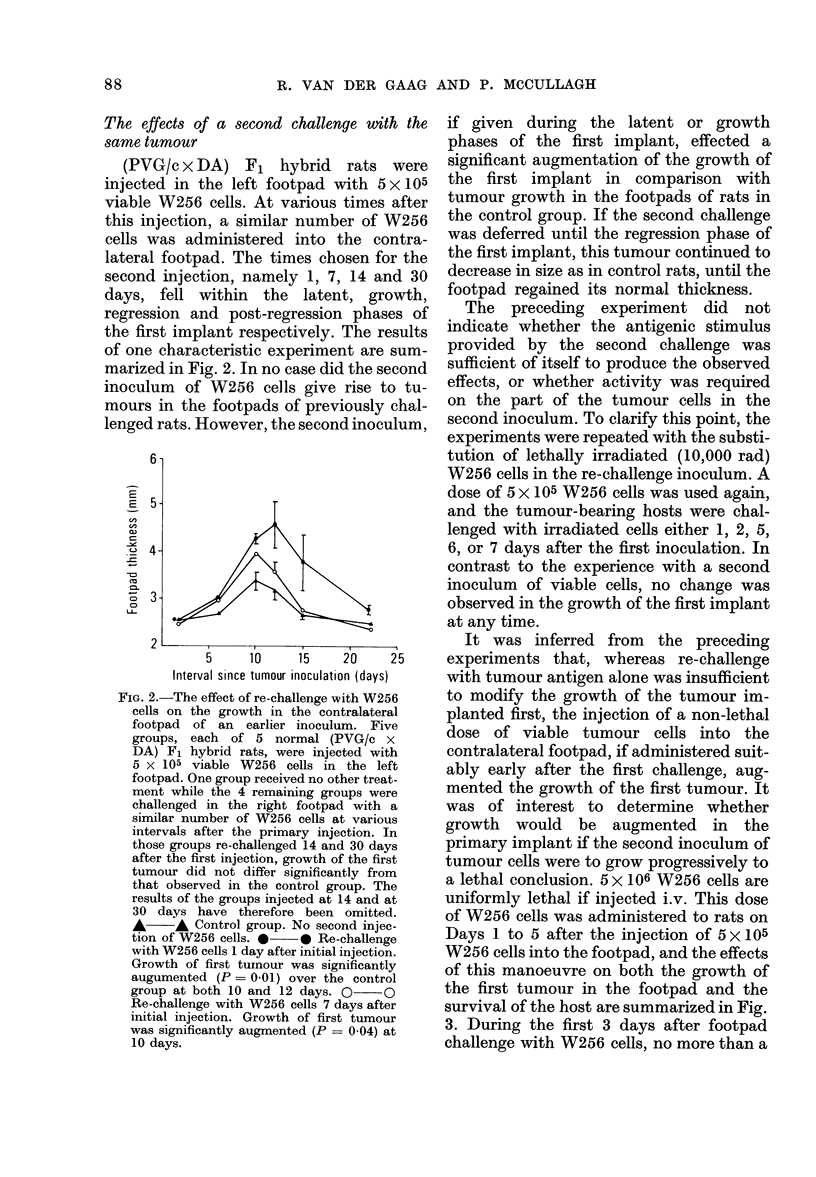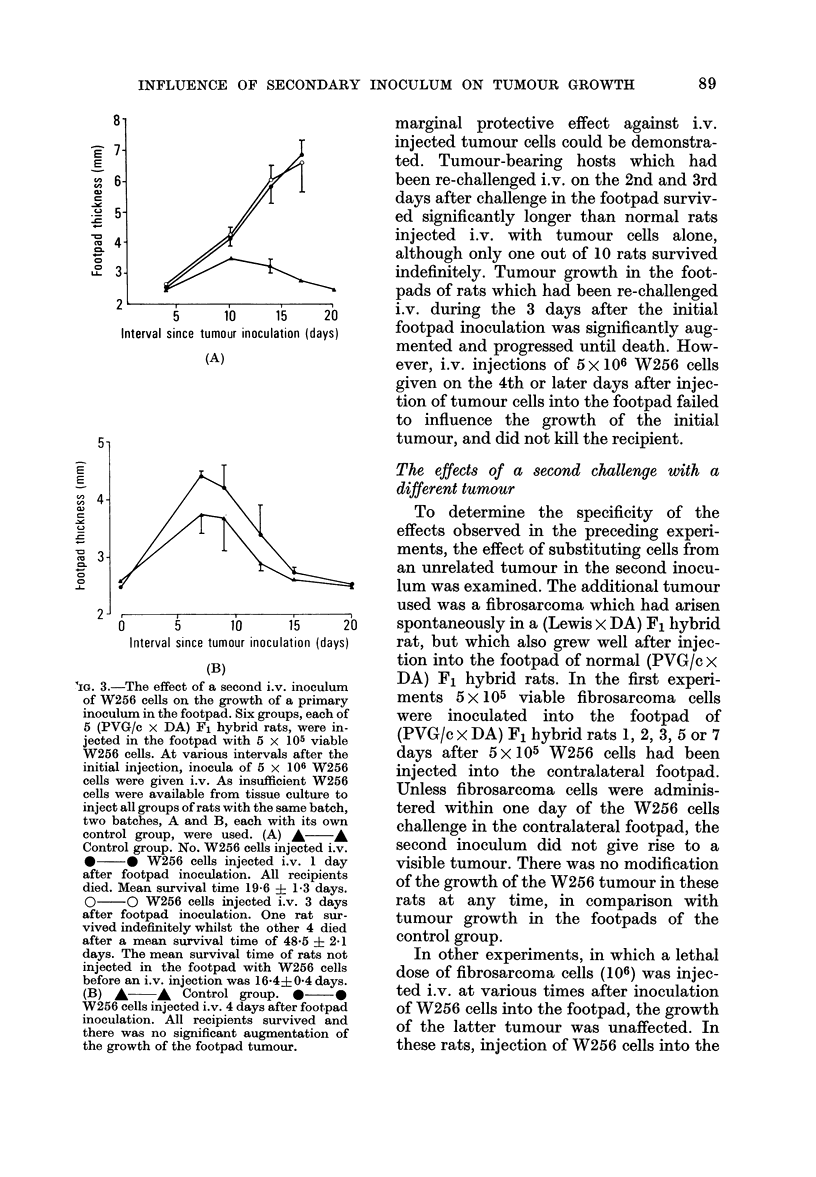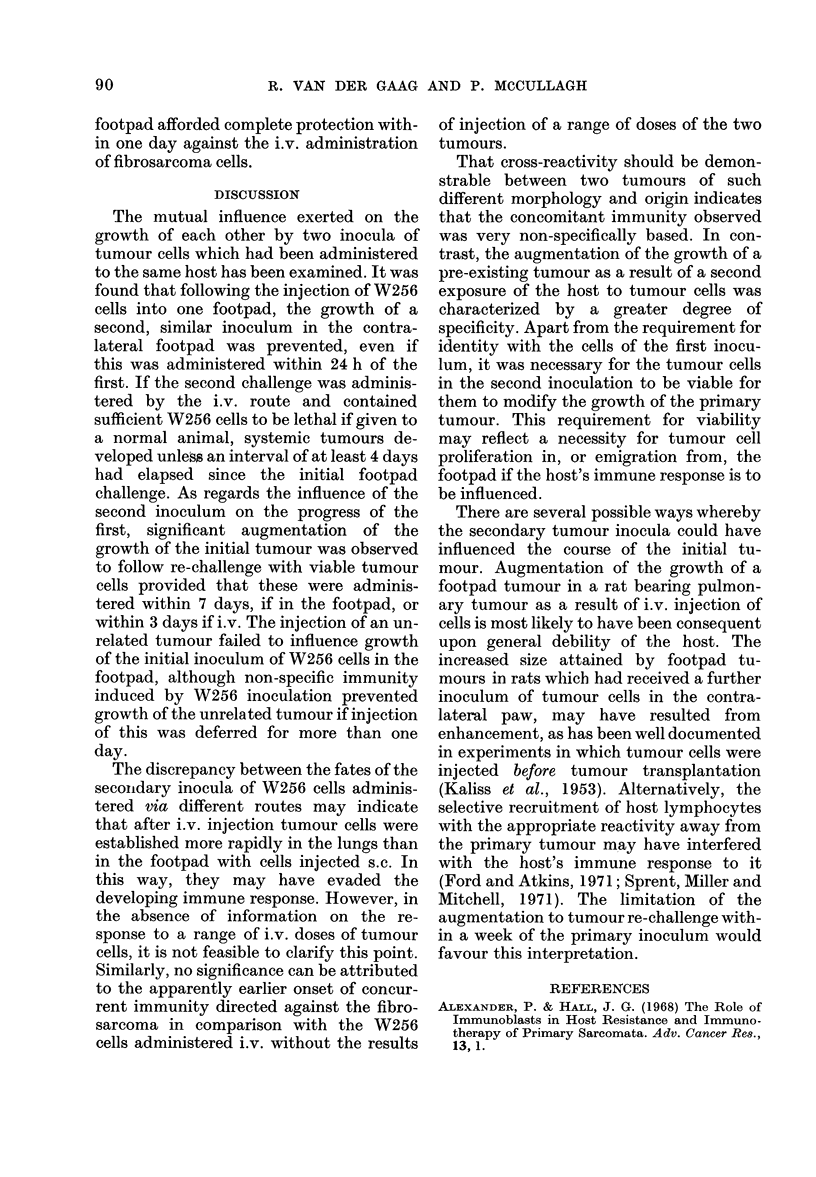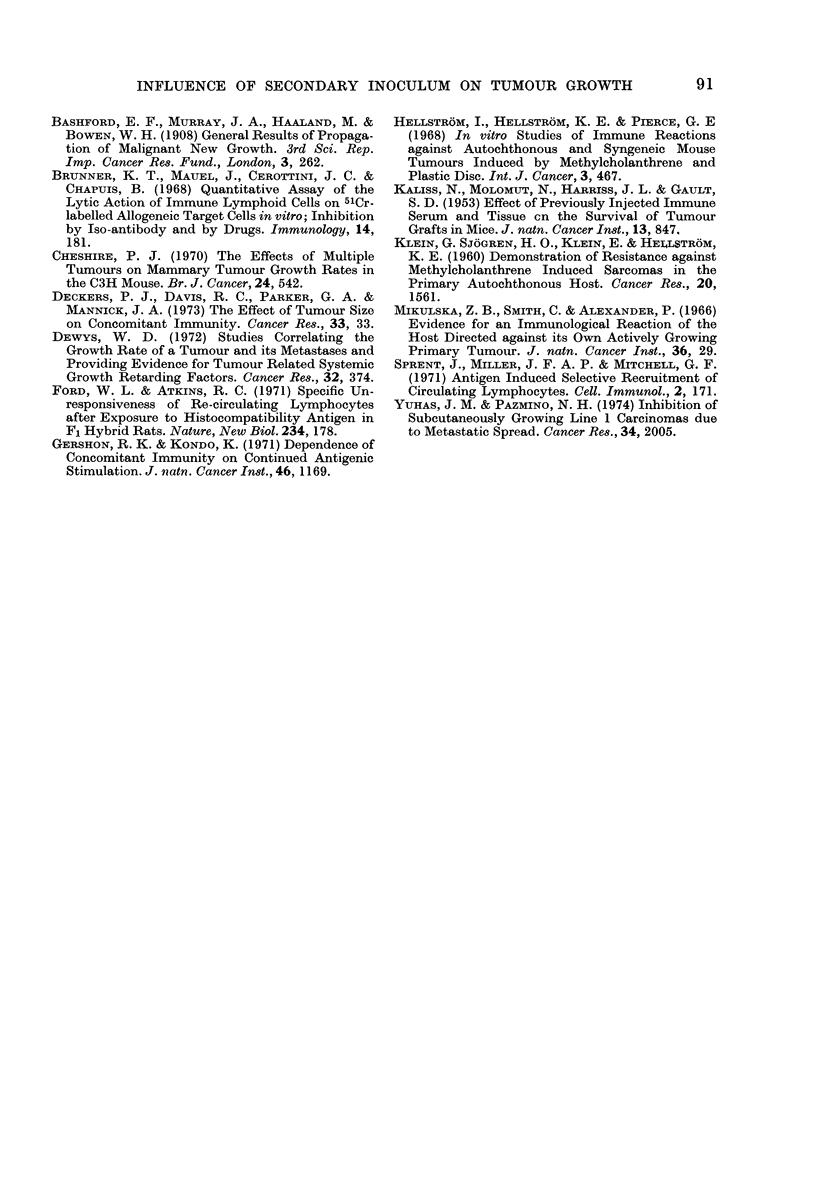# Influence of secondary inoculum of tumour cells on growth of primary tumour.

**DOI:** 10.1038/bjc.1978.13

**Published:** 1978-01

**Authors:** R. van der Gaag, P. McCullagh

## Abstract

The effect of successive inocula of tumour cells given to rats at intervals of 1 to 10 days was examined. If W256 cells were injected on both occasions, the second inoculum failed to grow if given into the footpad as early as 1 day, or intravenously as soon as 4 days, after the first administration. However, although a second inoculum failed to grow, it produced significant augmentation of the growth of the primary implant if given during its latent or growth phases. If the second inoculum contained cells from a fibrosarcoma unrelated to W256, its growth was effectively curtailed if the initial inoculum had preceded it by 24 h or more. However, secondary inocula of fibrosarcoma cells did not augment the growth of the primary W256 tumour.


*Br. J. Cancer* (1978) **37**, 86.

### INFLUENCE OF SECONDARY INOCULUM OF TUMOUR CELLS ON GROWTH OF PRIMARY TUMOUR

R. VAN DER GAAG* AND P. McCULLAGH

*From the Department of Immunology, John Curtin School of Medical Research, Australian National University, Canberra 2600, ACT, Australia*

Received 12 July 1977 Accepted 12 September 1977

**Summary.—The effect of successive inocula of tumour cells given to rats at intervals of 1 to 10 days was examined. If W256 cells were injected on both occasions, the second inoculum failed to grow if given into the footpad as early as 1 day, or intravenously as soon as 4 days, after the first administration. However, although a second inoculum failed to grow, it produced significant augmentation of the growth of the primary implant if given during its latent or growth phases. If the second inoculum contained cells from a fibrosarcoma unrelated to W256, its growth was effectively curtailed if the initial inoculum had preceded it by 24 h or more. However, secondary inocula of fibrosarcoma cells did not augment the growth of the primary W256 tumour.**

ALTHOUGH tumour-bearing hosts mount immune responses to both autochthonous and transplanted tumours, these responses are inadequate to prevent progressive growth (Alexander and Hall, 1968; Brunner *et al.*, 1968; Hellström, Hellström and Pierce, 1968; Klein *et al.*, 1960; Mikulska, Smith and Alexander, 1966). Whilst spontaneous regression of a well established primary tumour is infrequent, the host is often resistant both to secondary implants and to the establishment of metastases from the primary tumour, provided that growth of this is still in progress (Bashford *et al.*, 1908; Deckers *et al.*, 1973; Gershon and Kondo, 1971). Whilst the influence of a primary tumour on the establishment and growth of spontaneous or artificially induced secondary tumours has been thoroughly documented, there have been few investigations of the influence of the second implant on the growth of the primary. Yet this effect may be particularly relevant to clinical observations in humans, where an apparent change in the growth pattern of the primary tumour could be due to the establishment of metastases. Indeed reports of animal studies suggest that secondary tumours may produce considerable effects on the growth of the primary (Cheshire, 1970; Dewys, 1972; Yuhas and Pazmiño, 1974).

This paper reports the influence of a second challenge with cells from the same or a different tumour on the growth of a non-lethal tumour which had been inoculated previously. The first tumour can be inferred to have induced an immunological response as, after its regression, hosts are resistant to a second implant. The influence of the second challenge on the growth of the first implant, and the extent of immunity of the host towards a second challenge, were examined following re-administration of tumour cells at different sites during either the growth or regression phases of the first implant.

### MATERIALS AND METHODS

*Rats.*—Seven- to 9-week-old male and female (PVG/c $\times$ DA) $F_1$ hybrid rats were used.

* Present address: Central Laboratory, Netherlands Red Cross Bloodtransfusion Service, Postbus 9190, Amsterdam, The Netherlands.

In any individual experiment, rats of the same sex and age were used.

*Tumours.*—The Walker 256 carcinoma (W256) was obtained from Dr M. Cauchi, Monash University, Melbourne. W256 cells were cultured in 250ml Falcon plastic tissue-culture flasks in Medium F15 (Eagle's minimum essential medium, Gibco, U.S.A.) supplemented with 10% foetal calf serum and 100 u/ml Mycostatin. W256 cells were harvested in Puck's saline containing 0·025% trypsin and 0·01% versene. Tumour cells were washed twice in Hanks' balanced salt solution (HBSS) and their viability was established by the use of 0·1% trypan blue in saline. In order to avoid the preferential selection of certain cells under tissue culture conditions, new tumour-cell cultures were set up each month from a stock vial stored in liquid $N_2$.

The fibrosarcoma used arose spontaneously in a (Lewis × DA) $F_1$ hybrid male rat and was adapted to the same tissue-culture conditions as the Walker 256 carcinoma.

*Tumour inoculation.*—Recipients were anaesthetized with ether. Footpad injections (0·1–0·2 ml) were given into the subcutaneous tissue in the middle of the foot, the needle being inserted just distal to the heel. The lateral tail vein was used for i.v. injections (1–3 ml).

*Tumour growth measurement.*—Footpad size was measured on alternate days after tumour-cell inoculation. Anaesthetized rats were examined in random order using calipers (Dial Caliper, Mitutoyo, Japan) with an accuracy of 0·1 mm. On the first day after the injection of tumour cells in HBSS or HBSS alone, a small footpad swelling was observed. The swelling had, however, completely regressed by the 4th day in the saline-injected controls, the time at which the first experimental readings were taken. At the doses used here for i.v. injection, assessment of tumour growth was an all-or-none phenomenon, based on the death of the host due to complete replacement of lung tissue by tumour, or his survival.

### RESULTS

The injection of W256 cells into the s.c. tissues of the footpad initated localized tumour growth. The consequences of injecting a variety of doses of viable W256 cells into the footpad of normal (PVG/c × DA) $F_1$ hybrid rats are shown in Fig. 1. A dose of at least $5 \times 10^7$ cells was required to ensure a lethal outcome to tumour growth, following non-reversible metastatic spread to the popliteal lymph node, the leg muscles and finally the lungs. Following injection of non-lethal doses, the tumour growth pattern took the form of a latent

FIG. 1.—Changes in footpad thickness following the s.c. injection of various numbers of W256 cells.
Normal (PVG/c × DA) $F_1$ hybrid rats were injected in one hind footpad with the indicated number of viable W256 cells. Each group consisted of 5 recipients. ●——● $10^5$ cells; ▲——▲ $10^6$ cells; ○——○ $10^7$ cells; ■——■ $5 \times 10^7$ cells. Following this injection of tumour cells all recipients died. Mean survival time was 18·6 ± 3·3 days.

period varying in length from 4 to 7 days followed by a growth phase of about 7–10 days, when tumour cells could be seen in the draining lymph node, and then by a regression phase with concurrent disappearance of tumour cells from the lymph node. Inoculation of $5 \times 10^5$ cells resulted in tumour growth in the footpads of 100% of normal $F_1$ hybrid rats. As tumour growth rate varied with the batch of W256 cells, each experiment included a control group of rats which were inoculated in the footpad with W256 cells, but received no other treatment. Tumour growth in the footpads of the rats in the experimental groups was assessed in comparison with tumour growth in these control rats. For this reason no mean values could be calculated using data from experiments done on different days, and in the following paragraphs the results of one characteristic test are given for each experimental protocol.

*The effects of a second challenge with the same tumour*

(PVG/c × DA) $F_1$ hybrid rats were injected in the left footpad with $5 \times 10^5$ viable W256 cells. At various times after this injection, a similar number of W256 cells was administered into the contralateral footpad. The times chosen for the second injection, namely 1, 7, 14 and 30 days, fell within the latent, growth, regression and post-regression phases of the first implant respectively. The results of one characteristic experiment are summarized in Fig. 2. In no case did the second inoculum of W256 cells give rise to tumours in the footpads of previously challenged rats. However, the second inoculum,

FIG. 2.—The effect of re-challenge with W256 cells on the growth in the contralateral footpad of an earlier inoculum. Five groups, each of 5 normal (PVG/c × DA) $F_1$ hybrid rats, were injected with $5 \times 10^5$ viable W256 cells in the left footpad. One group received no other treatment while the 4 remaining groups were challenged in the right footpad with a similar number of W256 cells at various intervals after the primary injection. In those groups re-challenged 14 and 30 days after the first injection, growth of the first tumour did not differ significantly from that observed in the control group. The results of the groups injected at 14 and at 30 days have therefore been omitted. ▲——▲ Control group. No second injection of W256 cells. ●——● Re-challenge with W256 cells 1 day after initial injection. Growth of first tumour was significantly augumented ($P = 0{\cdot}01$) over the control group at both 10 and 12 days. ○——○ Re-challenge with W256 cells 7 days after initial injection. Growth of first tumour was significantly augmented ($P = 0{\cdot}04$) at 10 days.

if given during the latent or growth phases of the first implant, effected a significant augmentation of the growth of the first implant in comparison with tumour growth in the footpads of rats in the control group. If the second challenge was deferred until the regression phase of the first implant, this tumour continued to decrease in size as in control rats, until the footpad regained its normal thickness.

The preceding experiment did not indicate whether the antigenic stimulus provided by the second challenge was sufficient of itself to produce the observed effects, or whether activity was required on the part of the tumour cells in the second inoculum. To clarify this point, the experiments were repeated with the substitution of lethally irradiated (10,000 rad) W256 cells in the re-challenge inoculum. A dose of $5 \times 10^5$ W256 cells was used again, and the tumour-bearing hosts were challenged with irradiated cells either 1, 2, 5, 6, or 7 days after the first inoculation. In contrast to the experience with a second inoculum of viable cells, no change was observed in the growth of the first implant at any time.

It was inferred from the preceding experiments that, whereas re-challenge with tumour antigen alone was insufficient to modify the growth of the tumour implanted first, the injection of a non-lethal dose of viable tumour cells into the contralateral footpad, if administered suitably early after the first challenge, augmented the growth of the first tumour. It was of interest to determine whether growth would be augmented in the primary implant if the second inoculum of tumour cells were to grow progressively to a lethal conclusion. $5 \times 10^6$ W256 cells are uniformly lethal if injected i.v. This dose of W256 cells was administered to rats on Days 1 to 5 after the injection of $5 \times 10^5$ W256 cells into the footpad, and the effects of this manoeuvre on both the growth of the first tumour in the footpad and the survival of the host are summarized in Fig. 3. During the first 3 days after footpad challenge with W256 cells, no more than a

(A)

(B)

FIG. 3.—The effect of a second i.v. inoculum of W256 cells on the growth of a primary inoculum in the footpad. Six groups, each of 5 (PVG/c × DA) $F_1$ hybrid rats, were injected in the footpad with 5 × $10^5$ viable W256 cells. At various intervals after the initial injection, inocula of 5 × $10^6$ W256 cells were given i.v. As insufficient W256 cells were available from tissue culture to inject all groups of rats with the same batch, two batches, A and B, each with its own control group, were used. (A) ▲——▲ Control group. No. W256 cells injected i.v. ●——● W256 cells injected i.v. 1 day after footpad inoculation. All recipients died. Mean survival time 19·6 ± 1·3 days. ○——○ W256 cells injected i.v. 3 days after footpad inoculation. One rat survived indefinitely whilst the other 4 died after a mean survival time of 48·5 ± 2·1 days. The mean survival time of rats not injected in the footpad with W256 cells before an i.v. injection was 16·4±0·4 days. (B) ▲——▲ Control group. ●——● W256 cells injected i.v. 4 days after footpad inoculation. All recipients survived and there was no significant augmentation of the growth of the footpad tumour.

marginal protective effect against i.v. injected tumour cells could be demonstrated. Tumour-bearing hosts which had been re-challenged i.v. on the 2nd and 3rd days after challenge in the footpad survived significantly longer than normal rats injected i.v. with tumour cells alone, although only one out of 10 rats survived indefinitely. Tumour growth in the footpads of rats which had been re-challenged i.v. during the 3 days after the initial footpad inoculation was significantly augmented and progressed until death. However, i.v. injections of 5×$10^6$ W256 cells given on the 4th or later days after injection of tumour cells into the footpad failed to influence the growth of the initial tumour, and did not kill the recipient.

*The effects of a second challenge with a different tumour*

To determine the specificity of the effects observed in the preceding experiments, the effect of substituting cells from an unrelated tumour in the second inoculum was examined. The additional tumour used was a fibrosarcoma which had arisen spontaneously in a (Lewis×DA) $F_1$ hybrid rat, but which also grew well after injection into the footpad of normal (PVG/c× DA) $F_1$ hybrid rats. In the first experiments 5×$10^5$ viable fibrosarcoma cells were inoculated into the footpad of (PVG/c×DA) $F_1$ hybrid rats 1, 2, 3, 5 or 7 days after 5×$10^5$ W256 cells had been injected into the contralateral footpad. Unless fibrosarcoma cells were administered within one day of the W256 cells challenge in the contralateral footpad, the second inoculum did not give rise to a visible tumour. There was no modification of the growth of the W256 tumour in these rats at any time, in comparison with tumour growth in the footpads of the control group.

In other experiments, in which a lethal dose of fibrosarcoma cells ($10^6$) was injected i.v. at various times after inoculation of W256 cells into the footpad, the growth of the latter tumour was unaffected. In these rats, injection of W256 cells into the footpad afforded complete protection within one day against the i.v. administration of fibrosarcoma cells.

### DISCUSSION

The mutual influence exerted on the growth of each other by two inocula of tumour cells which had been administered to the same host has been examined. It was found that following the injection of W256 cells into one footpad, the growth of a second, similar inoculum in the contralateral footpad was prevented, even if this was administered within 24 h of the first. If the second challenge was administered by the i.v. route and contained sufficient W256 cells to be lethal if given to a normal animal, systemic tumours developed unless an interval of at least 4 days had elapsed since the initial footpad challenge. As regards the influence of the second inoculum on the progress of the first, significant augmentation of the growth of the initial tumour was observed to follow re-challenge with viable tumour cells provided that these were administered within 7 days, if in the footpad, or within 3 days if i.v. The injection of an unrelated tumour failed to influence growth of the initial inoculum of W256 cells in the footpad, although non-specific immunity induced by W256 inoculation prevented growth of the unrelated tumour if injection of this was deferred for more than one day.

The discrepancy between the fates of the secondary inocula of W256 cells administered *via* different routes may indicate that after i.v. injection tumour cells were established more rapidly in the lungs than in the footpad with cells injected s.c. In this way, they may have evaded the developing immune response. However, in the absence of information on the response to a range of i.v. doses of tumour cells, it is not feasible to clarify this point. Similarly, no significance can be attributed to the apparently earlier onset of concurrent immunity directed against the fibrosarcoma in comparison with the W256 cells administered i.v. without the results of injection of a range of doses of the two tumours.

That cross-reactivity should be demonstrable between two tumours of such different morphology and origin indicates that the concomitant immunity observed was very non-specifically based. In contrast, the augmentation of the growth of a pre-existing tumour as a result of a second exposure of the host to tumour cells was characterized by a greater degree of specificity. Apart from the requirement for identity with the cells of the first inoculum, it was necessary for the tumour cells in the second inoculation to be viable for them to modify the growth of the primary tumour. This requirement for viability may reflect a necessity for tumour cell proliferation in, or emigration from, the footpad if the host's immune response is to be influenced.

There are several possible ways whereby the secondary tumour inocula could have influenced the course of the initial tumour. Augmentation of the growth of a footpad tumour in a rat bearing pulmonary tumour as a result of i.v. injection of cells is most likely to have been consequent upon general debility of the host. The increased size attained by footpad tumours in rats which had received a further inoculum of tumour cells in the contralateral paw, may have resulted from enhancement, as has been well documented in experiments in which tumour cells were injected *before* tumour transplantation (Kaliss *et al.*, 1953). Alternatively, the selective recruitment of host lymphocytes with the appropriate reactivity away from the primary tumour may have interfered with the host's immune response to it (Ford and Atkins, 1971; Sprent, Miller and Mitchell, 1971). The limitation of the augmentation to tumour re-challenge within a week of the primary inoculum would favour this interpretation.

### REFERENCES

ALEXANDER, P. & HALL, J. G. (1968) The Role of Immunoblasts in Host Resistance and Immunotherapy of Primary Sarcomata. *Adv. Cancer Res.*, **13**, 1.

Bashford, E. F., Murray, J. A., Haaland, M. & Bowen, W. H. (1908) General Results of Propagation of Malignant New Growth. *3rd Sci. Rep. Imp. Cancer Res. Fund., London*, **3**, 262.

Brunner, K. T., Mauel, J., Cerottini, J. C. & Chapuis, B. (1968) Quantitative Assay of the Lytic Action of Immune Lymphoid Cells on $^{51}Cr$-labelled Allogeneic Target Cells *in vitro*; Inhibition by Iso-antibody and by Drugs. *Immunology*, **14**, 181.

Cheshire, P. J. (1970) The Effects of Multiple Tumours on Mammary Tumour Growth Rates in the C3H Mouse. *Br. J. Cancer*, **24**, 542.

Deckers, P. J., Davis, R. C., Parker, G. A. & Mannick, J. A. (1973) The Effect of Tumour Size on Concomitant Immunity. *Cancer Res.*, **33**, 33.

Dewys, W. D. (1972) Studies Correlating the Growth Rate of a Tumour and its Metastases and Providing Evidence for Tumour Related Systemic Growth Retarding Factors. *Cancer Res.*, **32**, 374.

Ford, W. L. & Atkins, R. C. (1971) Specific Unresponsiveness of Re-circulating Lymphocytes after Exposure to Histocompatibility Antigen in $F_1$ Hybrid Rats. *Nature, New Biol.* **234**, 178.

Gershon, R. K. & Kondo, K. (1971) Dependence of Concomitant Immunity on Continued Antigenic Stimulation. *J. natn. Cancer Inst.*, **46**, 1169.

Hellström, I., Hellström, K. E. & Pierce, G. E (1968) *In vitro* Studies of Immune Reactions against Autochthonous and Syngeneic Mouse Tumours Induced by Methylcholanthrene and Plastic Disc. *Int. J. Cancer*, **3**, 467.

Kaliss, N., Molomut, N., Harriss, J. L. & Gault, S. D. (1953) Effect of Previously Injected Immune Serum and Tissue cn the Survival of Tumour Grafts in Mice. *J. natn. Cancer Inst.*, **13**, 847.

Klein, G. Sjögren, H. O., Klein, E. & Hellström, K. E. (1960) Demonstration of Resistance against Methylcholanthrene Induced Sarcomas in the Primary Autochthonous Host. *Cancer Res.*, **20**, 1561.

Mikulska, Z. B., Smith, C. & Alexander, P. (1966) Evidence for an Immunological Reaction of the Host Directed against its Own Actively Growing Primary Tumour. *J. natn. Cancer Inst.*, **36**, 29.

Sprent, J., Miller, J. F. A. P. & Mitchell, G. F. (1971) Antigen Induced Selective Recruitment of Circulating Lymphocytes. *Cell. Immunol.*, **2**, 171.

Yuhas, J. M. & Pazmino, N. H. (1974) Inhibition of Subcutaneously Growing Line 1 Carcinomas due to Metastatic Spread. *Cancer Res.*, **34**, 2005.